# Evaluation of Dilution Susceptibility Testing Methods for Aztreonam in Combination with Avibactam against Enterobacterales

**DOI:** 10.1128/spectrum.03601-22

**Published:** 2022-11-07

**Authors:** C. J. Kelley, A. Kennedy-Mendez, O. N. Walser, M. T. Thwaites, F. F. Arhin, C. M. Pillar, D. A. Hufnagel

**Affiliations:** a Microbiologics Antibiotic and Microbiome Research Center, Kalamazoo, Michigan, USA; b Pfizer Canada, Kirkland, Quebec, Canada; University of North Carolina at Chapel Hill

**Keywords:** antibiotic resistance, beta-lactamases, clinical methods, Enterobacterales, metalloenzymes, monobactams, preclinical drug studies, susceptibility testing

## Abstract

As multidrug and pan-resistance among Enterobacterales continue to increase, there is an urgent need for more therapeutic options to treat these infections. New β-lactam and β-lactam inhibitor (BLI) combinations have a broad spectrum of activity, but those currently approved do not provide coverage against isolates harboring metallo-β-lactamases (MBL). Aztreonam (ATM) and avibactam (AVI) in combination (ATM/AVI; AVI at 4 μg/mL fixed concentration) provides a similarly broad range of activity while maintaining activity against MBL-producing isolates. The in vitro susceptibility testing of ATM/AVI by standard methods was evaluated during development. This study investigated the impact of nonstandard testing conditions on the activity of ATM/AVI as observed during broth microdilution testing as well as the equivalency between agar dilution and broth microdilution MIC values when testing a diverse panel of Enterobacterales (*N* = 201). Nonstandard test conditions evaluated included inoculum density, atmosphere of incubation, media pH, varied medium cation concentrations, incubation time, varied serum concentrations, testing in pooled urine instead of media, addition of blood to the media, and the presence of surfactant. Generally, apart from low pH and high inoculum density, nonstandard testing parameters did not affect ATM/AVI broth microdilution MIC values. Correlation of MIC values obtained by agar dilution and broth microdilution resulted in an essential agreement of 97.0% for all tested Enterobacterales. Variation of standard testing conditions had little impact on broth microdilution MIC values for ATM/AVI. The correlation between broth microdilution and agar dilution MICs suggests both methods are reliable for determination of ATM/AVI MIC values.

**IMPORTANCE** Increasing antibiotic resistance and emergence of pan-resistant isolates threaten the ability to control infections and to provide many other medical interventions such as surgery and chemotherapy, among others. New therapies are required to control emerging resistance mechanisms, including the increase in metallo-β-lactamases. Some new antibiotic combinations provide coverage against highly resistant isolates but are unable to target organisms that produce metallo-β-lactamases. Aztreonam in combination with avibactam provides a broad spectrum of activity against highly resistant isolates that also targets metallo-β-lactamase-producing organisms. An important part of drug development is the ability for clinical labs to determine the susceptibility of isolates to the antimicrobial. This manuscript investigates the in vitro susceptibility testing of aztreonam/avibactam with nonstandard testing conditions and a correlation study between broth microdilution and agar dilution against clinical isolates encoding a variety of resistance mechanisms. Overall, aztreonam/avibactam was generally unaffected by changes in testing conditions and showed strong agar/broth correlation.

## INTRODUCTION

With the increasing trends of antibiotic resistance, the development of therapies that target difficult-to-treat organisms with resistance mechanisms are of critical importance. As of 2019, an estimated 1.27 million global deaths were directly attributed to antimicrobial-resistant (AMR) infections and approximately 4.95 million deaths were associated with AMR (1). Models predict that deaths due to AMR will rise above 10 million by 2050, surpassing deaths due to cancer, in large part due to infections with no treatment options (2). An increasing number of clinical reports describe cases where no or few antibiotics were able to treat multidrug-resistant (MDR) isolates, including cases where infections were caused by isolates with no known susceptible results (pandrug-resistant) (3).

MDR Enterobacterales and carbapenem-resistant Enterobacterales (CRE) have garnered special attention in the research community due to their categorization as emerging threats by public health agencies (4, 5). Beta-lactam resistance is largely driven by the presence of beta-lactamases (BL) that disrupt the beta-lactam bond causing these antibiotics to be ineffective. There are extended-spectrum beta-lactamases (ESBL), carbapenemases, AmpC cephalosporinases, and MBL that all can protect Enterobacterales from various beta-lactams (6). AVI has an increased spectrum compared with other BLIs as it can inhibit AmpC, Klebsiella pneumoniae carbapenemase (KPC), and OXA-48 but is unable to inhibit MBL (6–9).

In 2015, coadministration of the cephalosporin ceftazidime with AVI (CAZ/AVI) was approved for treatment of infections caused by Enterobacterales and Pseudomonas aeruginosa (10). CAZ/AVI has become a leading choice for infections complicated by carbapenem resistance and effectively targets a broad range of Enterobacterales with KPCs and AmpC-type β-lactamases (11). CAZ/AVI was determined to be preferential for treatment of CRE infections based in part on its limited side effects when compared to colistin (12). However, the long-term viability of CAZ/AVI as a treatment option against CRE is challenged by isolates producing MBLs against which there are no clinically approved inhibitors (13). In the absence of an effective inhibitor, MBLs can hydrolyze CAZ, making isolates possessing MBLs resistant to this otherwise promising combination therapy (9). New Delhi metallo-beta-lactamase 1 (NDM-1) was first described in 2009 in Sweden from a patient that returned from a visit to India with a multidrug-resistant K. pneumoniae infection (14). MBLs were quite rare in North America prior to 2005; now NDMs are the second most common carbapenemase in Canada (15), and the CDC estimates that NDM-, Verona Integron-encoded MBL (VIM)-, and active-on-imipenem (IMP)-encoding isolates comprise ~10% of CRE infections in the United States (5). This highlights the growing need for treatment options that can cover this emerging resistance mechanism, either by discovery of new agents that are unaffected by these mechanisms or potentiating the utility of available beta-lactams by developing suitable β-lactam/BLI combinations.

In order to circumvent resistance to CAZ/AVI by MBL-producing Enterobacterales, though unapproved, clinicians have begun using aztreonam (ATM) in combination with CAZ/AVI (16, 17). The monobactam, ATM, is recalcitrant to hydrolysis of MBLs and retains its activity in their presence (18). As a result, the combination of ATM and AVI is active against ESBL- and KPC-producing strains and has the added benefit of being active against MBL-encoding isolates (19). The use of CAZ/AVI with ATM offered a therapeutic advantage with decreased mortality rate in bloodstream infections caused by MBL-producing Enterobacterales in comparison with treatment of patients with other agents (20). ATM is undergoing clinical development in combination with avibactam (ATM/AVI) for the treatment of serious infections caused by Gram-negative bacteria, including complicated intraabdominal infections (cIAI) and nosocomial pneumonia, including hospital-acquired pneumonia (HAP) and ventilator-associated pneumonia (VAP), and a separate trial involving patients with infections caused by Gram-negative bacteria with MBLs.

During development of a new antibiotic therapy, susceptibility testing methods must be evaluated so that throughout clinical development and postapproval there are reliable and reproducible methods for clinical and reference laboratories to use when testing clinical isolates. Currently, there are accepted Clinical and Laboratory Standards Institute (CLSI) quality control (QC) ranges for broth microdilution and disk diffusion susceptibility testing of ATM/AVI against relevant QC organisms including: Escherichia coli ATCC 25922, P. aeruginosa ATCC 27853 (AmpC), E. coli ATCC 35218 (TEM-1), and K. pneumoniae ATCC 700603 (SHV-18, OXA-2) (21, 22). In accordance with a Tier 1 evaluation of susceptibility testing methods for ATM/AVI as described by CLSI in M23 (23), the impact of nonstandard test parameters on broth microdilution ATM/AVI MIC values and the correlation between broth MIC and agar MIC values for ATM/AVI were evaluated and are summarized below.

## RESULTS

### Nonstandard broth microdilution testing conditions.

In this study, the impact of nonstandard test conditions on the activity of ATM/AVI in combination (AVI tested at a constant concentration of 4 μg/mL), ATM alone, AVI alone, and meropenem were evaluated. Test conditions evaluated included media pH, inoculum size, atmosphere of incubation, cation concentration of test media, time of incubation, serum concentration, testing in pooled urine at 2 different pHs, the addition of blood to the media, and the addition of surfactant to the media. The effect of these conditions on ATM and AVI monotherapy and combination therapy were evaluated against seven different isolates in this portion of the study comprising: two E. coli, four K. pneumoniae, and one P. aeruginosa QC organism.

The susceptibility testing data for the seven evaluated isolates is presented by organism in Table 1 (ATM/AVI) and Table S1 (meropenem, ATM alone, and AVI alone): E. coli ATCC 25922 (QC/non-MDR), E. coli NCTC 13353 (CTX-M-15), P. aeruginosa ATCC 27853 (QC/inducible AmpC), K. pneumoniae ATCC 700603 (SHV-18/OXA-2/OmpK35/OmpK37), K. pneumoniae ATCC BAA-1705 (KPC-2/TEM/SHV), K. pneumoniae CDC 0135 (VIM), and K. pneumoniae ATCC BAA-2146 (NDM). MIC values are shown grouped by test parameter, and the standard test condition for each evaluated parameter is highlighted in gray to facilitate comparison of MIC values at nonstandard conditions relative to standard conditions (evaluated in parallel for each test parameter). The MIC values of ATM/AVI, meropenem, and ATM alone under standard conditions were within the established CLSI QC range on all dates tested for E. coli ATCC 25922, P. aeruginosa ATCC 27853, and K. pneumoniae ATCC 700603. Under standard conditions across the evaluated organisms that produce beta-lactamases, the activity of ATM increased when tested in combination with AVI. These results demonstrate the anticipated synergy between ATM and AVI in combination for the beta-lactamase producing isolates.

**TABLE 1:** MIC values of aztreonam in combination with avibactam when tested under standard and nonstandard conditions against seven organisms

Conditions	MIC (μg/mL)						
	Aztreonam						
Isolate	E. coli ATCC 25922	E. coli NCTC 13353	K. pneumoniae ATCC 700603	K. pneumoniae ATCC BAA-1705	K. pneumoniae CDC 0135	K. pneumoniae ATCC BAA-2146	P. aeruginosa ATCC 27853
pH 5.0	0.25	64	32	>64	>64	>64	4
pH 6.0^*a*^	0.12	64	64	>64	>64	>64	2
pH 7.4	≤0.06 (0.06 to 0.25)	64	64 (8 to 64)	>64	>64	>64	2 (2 to 8)
pH 8.0	≤0.06	64	16	>64	>64	64	2
Inoculum −1.1 × 10^4^ CFU/mL	0.12	64	16	>64	>64	64	4
Inoculum −1.1 × 10^5^ CFU/mL	0.12 (0.06 to 0.25)	64	32 (8 to 64)	>64	>64	>64	4 (2 to 8)
Inoculum −1.1 × 10^6^ CFU/mL	>64#	>64	>64#	>64	>64	>64	64^*b*^
Inoculum −1.1 × 10^7^ CFU/mL	>64#	>64	>64#	>64	>64	>64	>64^*b*^
Ambient atmosphere	≤0.06 (0.06 to 0.25)	64	64 (8 to 64)	>64	>64	>64	2 (2 to 8)
5% CO_2_ atmosphere	≤0.06	>64	64	>64	>64	>64	1
MHB w/25 Ca^2+^/12.5 Mg^2+^ (mg/L)	0.12 (0.06 to 0.25)	64	32 (8 to 64)	>64	>64	64	8 (2 to 8)
MHB w/5 Ca^2+^/5 Mg^2+^ (mg/L)	0.12	64	32	>64	>64	64	4
MHB w/25 Ca^2+^/5 Mg^2+^ (mg/L)	0.12	64	32	>64	>64	64	4
MHB w/5 Ca^2+^/12.5 Mg^2+^ (mg/L)	0.12	64	32	>64	>64	64	4
MHB w/50 Ca^2+^/25 Mg^2+^ (mg/L)	0.12	64	32	>64	>64	>64	4
18-h incubation	≤0.06 (0.06 to 0.25)	64	64 (8 to 64)	>64	>64	>64	2 (2 to 8)
24-h incubation	≤0.06	64	64	>64	>64	>64	2
48-h incubation	≤0.06	64	64	>64	>64	>64	2
No human serum/albumin	0.12 (0.06 to 0.25)	64	32 (8 to 64)	>64	>64	>64	2 (2 to 8)
10% human serum	0.12	64	32	NG	>64	64	2
50% human serum	≤0.06	16	16	NG	>64	64	1#^*b*^
4% human serum albumin	0.25	64	64	>64	>64	>64	4
Urine (pH 6.8)	≤0.06	64	32	>64	>64	64	0.25#
Urine (pH 7.2)	≤0.06	8	16	>64	64	64	0.12#
CAMHB (pH 7.2)	≤0.06 (0.06 to 0.25)	64	64 (8 to 64)	>64	>64	>64	2 (2 to 8)
CAMHB (pH 6.8)	≤0.06	64	64	>64	>64	>64	2
3% lysed horse blood	≤0.06	64	64	>64	>64	64	1#
0.002% P-80	≤0.06	>64	32	>64	>64	>64	1#
No supplementation	≤0.06 (0.06 to 0.25)	64	64 (8 to 64)	>64	>64	>64	2 (2 to 8)

a Cells shaded gray represent MIC values as observed under standard conditions. CLSI QC ranges shown in parentheses where applicable.

b #, denotes where an MIC value fell outside the CLSI acceptable QC range in a condition for that particular isolate.

### Atmosphere of incubation, divalent cation concentration, and the presence of surfactant/blood.

There was no apparent impact on the activity of ATM, AVI, ATM in combination with AVI, or for meropenem across evaluated organisms by: incubation in the presence of CO_2_, divalent cation concentration, or the presence of LHB/P-80 (Table 1; Table S1).

### Media pH.

The antibacterial activity of ATM/AVI decreased when tested in media at low pH (pH 5.0) relative to standard pH when tested against E. coli ATCC 25922, P. aeruginosa ATCC 27853, and K. pneumoniae ATCC 700603. The activity of ATM in combination with AVI was generally not altered against the other four tested isolates. AVI monotherapy antibacterial activity was decreased at low pH for all isolates, excluding P. aeruginosa ATCC 27853, where the impact could not be evaluated due to inactivity of AVI alone (AVI MIC values >128 μg/mL). ATM activity alone was decreased at low pH only when testing against E. coli ATCC 25922 (Table 1; Table S1).

### Inoculum size.

At inoculum densities of approximately 5 × 10^7^ CFU/mL across the evaluated organisms, there was a noted decrease in activity of ATM alone, AVI alone, and ATM and AVI in combination relative to the standard inoculum density of approximately 5 × 10^5^ CFU/mL across the evaluated organisms. This increase was also apparent with ATM and/or AVI alone for select isolates at inoculum densities of approximately 5 × 10^6^ CFU/mL. This negative impact of increased inoculum size on activity was also apparent with meropenem for meropenem-susceptible isolates (Table 1; Table S1).

### Incubation time.

AVI alone had increased MIC values when evaluating longer incubation periods versus the standard duration of time for the evaluated organisms. ATM alone was unaffected by incubation length. ATM/AVI showed increased MIC values only against E. coli ATCC 25922 at the 48-h incubation time, while the six other evaluated isolates were unaffected by longer incubation times (Table 1; Table S1).

### Activity in the presence of human serum/albumin.

The presence of human serum and albumin did not affect the activity of ATM and AVI alone or in combination, with the exception of 50% human serum. The addition of 50% human serum caused a decrease in the MIC values of ATM and AVI in combination and AVI alone when evaluated against K. pneumoniae ATCC BAA-2146 and caused an increase in the AVI alone MIC value against K. pneumoniae CDC 0135. Of note, K. pneumoniae ATCC BAA-1705 was unable to grow in 10% or 50% human serum (Table 1; Table S1).

### Activity in urine.

Across the evaluated organisms (Table 1), ATM/AVI showed increased antibacterial activity when testing in urine relative to standard CAMHB though the magnitude of the increased activity varied by isolate. The activity of meropenem was largely unaffected by testing in urine in all isolates. ATM activity alone was also increased in urine for P. aeruginosa ATCC 27853, K. pneumoniae ATCC 700603 (at pH 7.2), and E. coli NCTC 13353 (pH 7.2) (Table S1).

### Correlation of broth microdilution and agar dilution MIC values.

The correlation between MIC values of ATM/AVI as determined by broth microdilution relative to MIC values determined by agar dilution is summarized below. ATM was included as the comparator.

**FIG 1:**
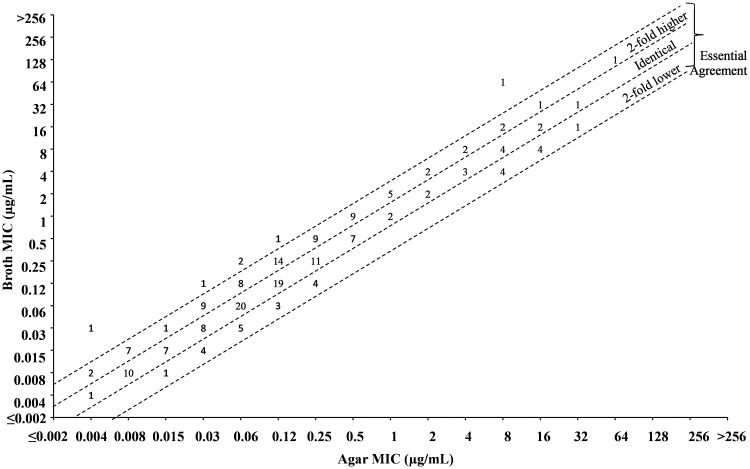
Comparison of aztreonam/avibactam broth and agar MIC values against Enterobacterales (*N* = 201) scatterplot. Reported MIC represents the aztreonam concentration of the combination (avibactam tested at constant concentration of 4 μg/mL by both methods).

**FIG 2:**
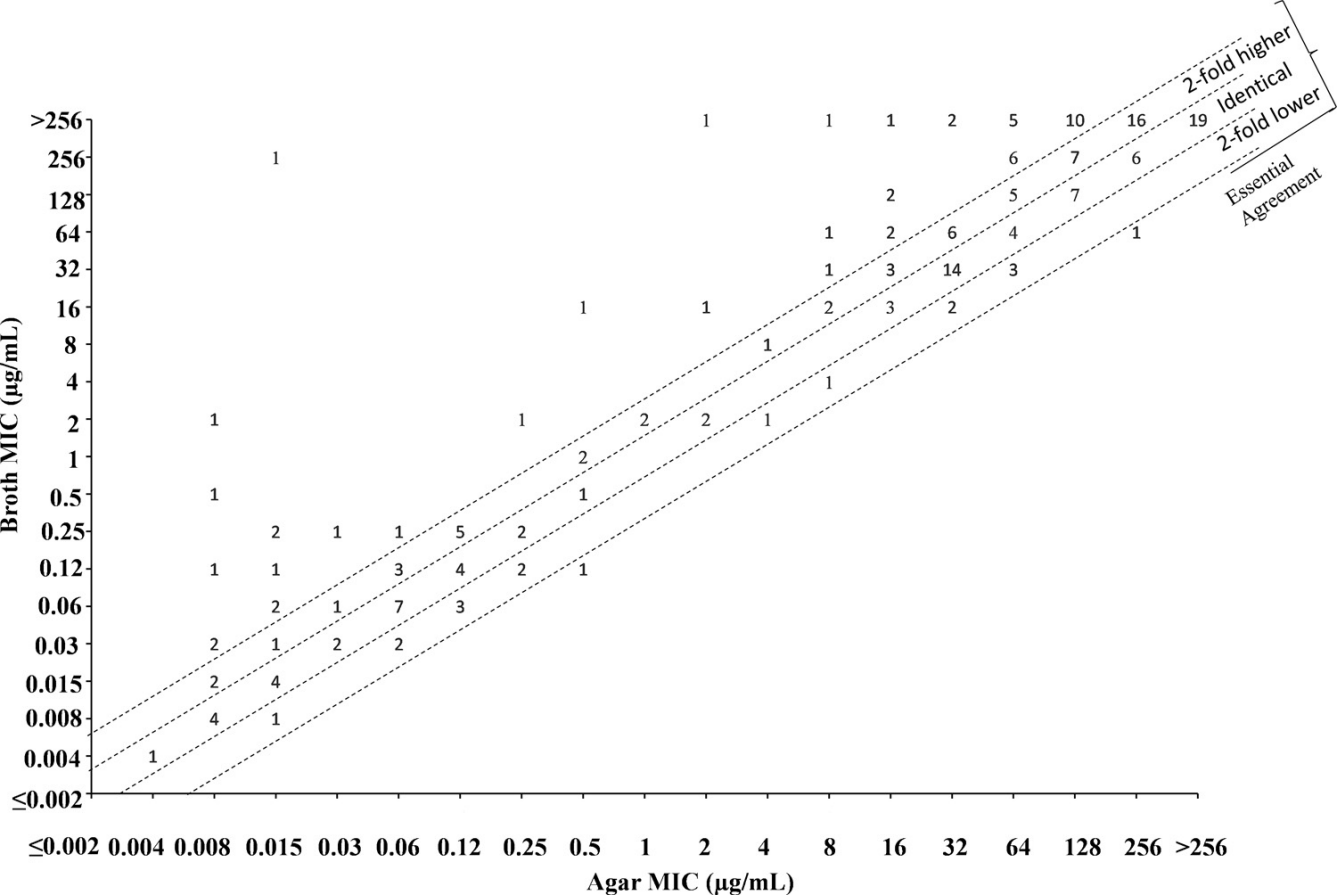
Comparison of aztreonam broth and agar MIC values against Enterobacterales (*N* = 201) scatterplot.

A summary of the overall essential agreement between the agar dilution and broth microdilution methods for ATM/AVI and ATM alone is shown in Table 2, and scatterplots comparing MIC values by method are shown in Fig. 1 and 2. Individual MIC values and details on beta-lactamase content among the tested isolates can be found in Table S2. All MIC values for ATM/AVI and ATM during this study were within the established CLSI QC ranges for the designated routine QC organisms. One replicate of E. coli ATCC 25922 was out of QC by broth microdilution for ATM/AVI; however, this organism is not a routine QC organism for the testing of this combination and the results on the same day of testing were in QC with the routine QC organism K. pneumoniae ATCC 700603 (Table S3).

**TABLE 2:** Comparison of MIC values for aztreonam/avibactam and aztreonam alone as determined concurrently by broth microdilution and agar dilution susceptibility testing against Enterobacterales

Aztreonam/avibactam										
			Fold-difference—broth MIC vs. agar MIC							
Organism	N^*a*^	EA^*b*^	≥–8	–4	–2	0	+2	+4	≥+4	≥+8
Enterobacterales	201	97.0%			26	97	72	4		2
E. coli	57	96.5%			12	23	20	1		1
K. pneumoniae	48	97.9%			4	26	17			1
Proteus spp.	20	100.0%			2	10	8			
E. cloacae	29	93.1%			2	12	13	2		
*Citrobacter* spp.	18	100.0%			2	12	4			
Other	29	96.6%			4	14	10	1		

Aztreonam										
			Fold-difference—broth MIC vs. agar MIC							
Organism	N^*a*^	EA^*b*^	≥–8	–4	–2	0	+2	+4	≥+4	≥+8
Enterobacterales	164	68.9%	1	2	15	60	38	14	15	19
E. coli	44	77.3%		1	5	13	16	4	5	
K. pneumoniae	35	82.9%			3	16	10	4	2	
Proteus spp.	20	55.0%			1	6	4	2		7
E. cloacae	23	60.9%			1	10	3		7	2
*Citrobacter* spp.	17	58.8%				7	3	1	1	5
Other	27	63.0%	1	1	5	8	4	3		5

a Number of evaluable isolates (isolates for which the fold-difference as indicated could be determined based on the MIC).

b EA, essential agreement (MIC value identical or within 2-fold between methods; shaded in gray).

Essential agreement between the methods was defined as having MIC values by both methods that were either identical or within 2-fold of each other. In initial screening there were 21 isolates that had a >2-fold difference between broth and agar MIC values; 10 of which differed by 4-fold. To address this, discordant isolates were run an additional four times with separate inocula. The median MIC value from the five individual datapoints was used as the final MIC value for these isolates during analysis of essential agreement and correlation between methods (Table S4). There was a high degree of essential agreement between both methods when testing ATM/AVI against Enterobacterales, with 97.0% essential agreement overall and ≥93.1% essential agreement by species (Table 2). Out of the resulting six discordant events, four were due to 4-fold differences between the methods with a trend toward higher MIC values by broth relative to agar. For the isolates that fell outside the essential agreement for ATM/AVI, no trends were observed for species type or BL type. In initial screening against Providencia rettgeri, broth and agar MIC values had large differences (broth:agar ratios of 4,096 or 8,192) in two out of the three tested isolates. To ensure this was not a genus specific issue with testing, an additional 21 *Providencia* underwent broth and agar MIC comparison for ATM/AVI and ATM alone (Table S5). Heavy trailing endpoints in the broth MIC assay that were not apparent by agar dilution were seen for both ATM and ATM/AVI when testing *P. rettgeri* (Fig. S1); the same was not an issue with P. stuartii. MIC values in broth for *P. rettgeri* were recorded where heavy trailing began, and MIC value determination by this method led to no instances of discordance in the 21 additional *Providencia* that were investigated (Table S5).

Discordance between broth and agar MIC values was apparent with ATM alone for many of the evaluated isolates, particularly in ATM-resistant isolates that harbored BLs, as evidenced by the comparatively lower rate of essential agreement between methods for ATM alone (68.9%) relative to the ATM/AVI combination (97.0%) (Table 2; Fig. 1 and 2).

## DISCUSSION

ATM in combination with AVI provides a new therapeutic option against MBL-producing Enterobacterales. A CDC study found that over 85% of MBL-producing isolates were susceptible to ATM/AVI, compared with 0% for CAZ/AVI and ~6% for ATM alone (24). With increasing trends of MBLs in North America and around the globe (5, 15), new therapeutic options are needed. In order for ATM/AVI to be properly utilized and administered to patients, clinical laboratories need to be able to perform susceptibility testing using standard methods. This study evaluated the impact of alterations to the standard method (e.g., increased inoculum density, altered medium pH, etc.) on the perceived activity of AVI/ATM and how well agar dilution MIC values correlated with broth microdilution MIC values.

The activity of ATM, AVI, or the combination of both was largely unaffected by alterations to the standard testing parameters. Atmospheric conditions, divalent cation concentrations, incubation time, the presence of human serum, activity in urine, and the presence of surfactant or blood did little to alter the activity of ATM, AVI, or their combination except in isolated cases (Table 1; Table S1). While test media pH generally did not affect MIC values, test media at pH-5 did decrease the activity of ATM/AVI for the majority of isolates. Inoculum density 2-logs higher than the standard resulted in decreased antibacterial activity of ATM, AVI, ATM/AVI, and meropenem, which is a common phenomenon when testing β-lactam antibiotics (Table 1; Table S1) (25). Interestingly, there was a trend toward increased activity of the combination of ATM and AVI when evaluated in urine (Table 1). Overall, ATM/AVI displayed similar antibacterial activity across many different testing conditions against seven different isolates which included those with diverse beta-lactamases. These results demonstrate that MIC values for the combination as determined with the standard broth microdilution method are stable and reliable assuming inoculum density and media pH is controlled.

The essential agreement between broth microdilution and agar dilution methods was high for the susceptibility testing of ATM/AVI (97.0%) and surpassed the >90% cited in CLSI M23 (23) required to establish equivalency between test methods. While endpoints were generally easy to interpret for ATM/AVI broth microdilution, P. rettgeri broth endpoints were read where heavy trailing began. It is important to note, if broth MIC values reported for P. rettgeri were based on complete inhibition, a large degree of discordance would have been apparent with broth MIC values being several-fold higher than those observed by agar dilution. Importantly, for many isolates where discordance was recorded between broth MIC values and agar MIC values for ATM, it was typically associated with a heavy trailing endpoint in the broth MIC assay, in particular with Citrobacter spp. and Proteus spp., similar to that seen with P. rettgeri for the combination.

The ATM/AVI combination under development can effectively target MBL-encoding Enterobacterales while retaining a spectrum of activity similar to CAZ/AVI against non-MBL CRE (19). ATM/AVI already has established CLSI broth microdilution QC ranges and disk diffusion QC ranges for four QC organisms with K. pneumoniae ATCC 700603 the recommended routine QC organism (21). The activity in the broth microdilution assay is largely unaffected by nonstandard testing parameters. Moreover, the essential agreement between agar dilution and broth microdilution MIC values exceeded minimum requirements established by CLSI (23). Overall, susceptibility testing of ATM/AVI by both broth microdilution and agar dilution methods when following CLSI guidelines using the established QC ranges provides reliable means for the dilution susceptibility testing of this combination throughout clinical development, surveillance, and at clinical laboratories postapproval.

## MATERIALS AND METHODS

### Test compounds.

AVI was provided by Pfizer (catalog no. PF-06416494-02; lot no. PF-06416494-02-0011) or GSK (lot no. G322044) and stored at 4°C. ATM (catalog no. 1046205; lot no. R08590) and meropenem (catalog no. 1392454; lot no. J0K434) were supplied by United States Pharmacopeia (USP; Rockville, MD) and were stored at −20°C and 4°C, respectively. AVI and meropenem were solubilized and diluted in water. ATM was solubilized in 10% sodium bicarbonate solution and diluted in water in accordance with guidelines from the CLSI (21, 22).

### Test organisms.

The test organisms evaluated in this study consisted of nonduplicate, nonconsecutive clinical isolates from the Micromyx Repository (MMX; Kalamazoo, MI) and from International Health Management Associates (IHMA; Schaumburg, IL), and reference isolates from the American Type Culture Collection (ATCC; Manassas, VA), the National Collection of Type Cultures (NCTC; Salisbury, UK), isolates, and the Centers for Disease Control and Prevention Antibiotic Resistance Bank (CDC; Atlanta, GA). Prior to testing, isolates were streaked from frozen stocks onto commercially prepared trypticase soy agar (TSA) with or without 5% sheep blood and were incubated at 35°C overnight. Isolates were selected to account for many different resistance phenotypes, including but not limited to, various BLs and MBLs. Table S1 provides BL content information on all of the isolates where available.

### Broth microdilution MIC assay (standard format).

MIC values were determined using a broth microdilution procedure described by CLSI (21, 22). The test medium used was Cation-Adjusted Mueller-Hinton Broth (CAMHB; BD, Sparks, MD; Lot No. 9239528, 9324795) with and without AVI at a final test concentration of 4 μg/mL. Media preparation, liquid handling, incubation, and panel reading were all performed as previously described (26, 27).

### Broth microdilution MIC assay (nonstandard conditions).

Nonstandard test parameters were evaluated in parallel with standard parameters during the study as described below:

**(i) pH.** The effect of test medium pH was assessed at pH 5.0, 6.0, 7.4 (reference pH), and 8.0. CAMHB was prepared and adjusted to the target pH.

**(ii) Inoculum effect.** An initial 0.5 McFarland cell suspension (approximately 1 to 2 × 10^8^ CFU/mL) was prepared and diluted to achieve final inoculum concentrations targeting 5 × 10^4^, 5 × 10^5^ (reference inoculum), 5 × 10^6^, and 5 × 10^7^ CFU/mL in the assay. Cell densities were determined by serial 10-fold dilution and track-dilution plating 10 μL onto TSA. Plates were angled to allow for the cell suspension to track down the agar surface. After cell suspensions had dried on the agar surface, plates were inverted and incubated at 35°C overnight, prior to enumeration of CFU.

**(iii) Atmosphere of incubation.** Assays were conducted in ambient atmosphere (standard atmosphere) and in the presence of approximately 5% CO_2_ using standard media.

**(iv) Cation supplementation.** The effect of cations was determined by assaying the compounds in unadjusted Mueller-Hinton Broth (MHB; BD, Sparks, MD; Lot No. 9156832) supplemented to 5 mg/L Ca^2+^ and 5 mg/L Mg^2+^, 25 mg/L Ca^2+^ and 5 mg/L Mg^2+^, 5 mg/L Ca^2+^ and 12.5 mg/L Mg^2+^, 50 mg/L Ca^2+^ and 25 mg/L Mg^2+^, as well as MHB supplemented to the standard 25 mg/L Ca^2+^ and 12.5 mg/L Mg^2+^ (reference cation supplementation). MHB was supplemented to result in the final Ca^2+^ and Mg^2+^ concentrations listed above by using a 10 mg/mL stock of MgCl_2_ and 10 mg/mL stock of CaCl_2_, each of which was prepared in accordance with CLSI guidelines (21, 22).

**(v) Incubation time.** Standard incubation time for the evaluated organisms is 16 h to 20 h. The effect of prolonged incubation time on MIC values was determined by reading MIC values following incubation for 18 h (standard), 24 h, and 48 h in standard test media.

**(vi) Human serum and serum albumin supplementation.** Assays were conducted in standard media in the presence and absence of pooled human serum and human serum albumin. Filter-sterilized pooled human serum (Innovative Research, Novi, MI; Cat. No. ISERABHI100ML; Lot No. 31436) was added at a final concentration of 10% and 50% (vol/vol) to CAMHB. Human serum albumin (Millipore Sigma, Billerica, MA; Cat. No. 12666; Lot No. 3386002) was added at a final concentration of 4% (wt/vol) to CAMHB and filter-sterilized before use in the assay.

**(vii) Media supplements.** The impact of testing in media containing 3% lysed horse blood (LHB; Hemostat; Dixon, CA; Lot No. 496003) and media containing 0.002% polysorbate-80 (P-80; Alfa Aesar; Ward Hill, MA; Ref. No. L13315; Lot No. 10197649) were evaluated relative to standard medium.

**(viii) Urine.** The impact of incubation in pooled normal human urine (Innovative Research, Novi, MI; Cat. No. ISERABHI100ML; Lot No. 32739) and pooled normal urine adjusted to neutral pH (7.2 to 7.4) was evaluated when compared with CAMHB at pH 7.2 to 7.4, as well as CAMHB adjusted to the pH of the pooled normal human urine (pH 6.8, as measured upon receipt). All solutions were filter-sterilized prior to use in the assay.

### Agar dilution MIC assay.

MIC values were determined using the agar dilution method (21, 22). All serial dilutions and liquid handling were performed by hand using sterile pipettes. ATM was prepared and diluted according to CLSI guidelines (21).

ATM was mixed with molten (50°C to 55°C) Mueller-Hinton agar (MHA; BD; Lot No. 8024807) with and without 4 μg/mL of AVI (final concentration in the assay). ATM was added in a ratio of 3 mL 10× test agent to 27 mL agar. Once test agents were added to the agar in a sterile tube, they were mixed gently and then poured into a sterile square petri plate (100 mm × 100 mm). Plates were allowed to solidify at room temperature and placed in a laminar airflow hood with the covers off to remove condensed moisture on the agar surface.

Using the same inoculum used for broth microdilution MIC testing (the 1:10 diluted 0.5 McFarland suspension described above), each bacterial cell suspension was then transferred to wells in a stainless-steel replicator block. The prongs on the replicator deliver approximately 1 to 2 μL of inoculum to an agar surface. The resulting inoculum spots contained approximately 10^4^ cells.

Each agar plate containing either drug or no drug (control) was stamped with a stainless-steel Steer’s replicator. All plates were placed on benchtop with the agar surface face up to allow for the inoculum to soak into the agar. The plates were inverted and incubated at 35°C for 20 h. The MIC was defined as the lowest test agent concentration that completely inhibited bacterial growth on the agar surface. Agar and broth MIC values were compared, and essential agreement was determined by grouping the percent of isolates that had a broth MIC within 2-fold of the agar MIC. Discordant ATM/AVI isolates were repeated an additional 4× to arbitrate MIC values.
