# Supplementary material for: Evaluation of Dilution Susceptibility Testing Methods for Aztreonam in Combination with Avibactam against Enterobacterales
Source: Microbiol Spectr. 2022 Nov 7;10(6):e03601-22. doi: 10.1128/spectrum.03601-22 (PMC9769647; doi:10.1128/spectrum.03601-22)

Supplemental Table 1. MIC Values of Meropenem, Aztreonam, and Avibactam When Tested Under Standard and Non-Standard Conditions Against 7 organisms

Conditions	MIC (µg/mL)						
	Meropenem						
Isolate	*E. coli* ATCC 25922	*E. coli* NCTC 13353	*K. pneumoniae* ATCC 700603	*K. pneumoniae* ATCC BAA-1705	*K. pneumoniae* CDC 0135	*K. pneumoniae* ATCC BAA-2146	*P. aeruginosa* ATCC 27853
pH 5.0	0.06	0.06	0.06	4	0.06	1	0.25
pH 6.0	0.03	0.03	0.03	>4	1	>4	0.12
pH 7.4	0.016 (0.008-0.06)	0.03	0.016	>4	2	>4	0.25 (0.12-1)
pH 8.0	0.008	0.03	0.03	>4	2	>4	0.5
Inoculum – 1.1 x 10^4 CFU/mL	0.016	0.03	0.016	2	2	>4	0.25
Inoculum – 1.1 x 10^5 CFU/mL	0.016 (0.008-0.06)	0.03	0.016	>4	>4	>4	1 (0.12-1)
Inoculum – 1.1 x 10^6 CFU/mL	0.03	0.03	0.03	>4	>4	>4	1
Inoculum – 1.1 x 10^7 CFU/mL	4#	2	4	>4	>4	>4	2#
ambient atmosphere	0.016 (0.008-0.06)	0.03	0.016	>4	2	>4	0.25 (0.12-1)
5% CO_2 atmosphere	0.016	0.016	0.016	>4	4	>4	0.12
MHB w/ 25 Ca^{2+}/12.5 Mg^{2+} (mg/L)	0.016 (0.008-0.06)	0.016	0.03	>4	2	>4	0.25 (0.12-1)
MHB w/ 5 Ca^{2+}/5 Mg^{2+} (mg/L)	0.016	0.03	0.03	>4	2	>4	0.25
MHB w/ 25 Ca^{2+}/5 Mg^{2+} (mg/L)	0.016	0.03	0.016	>4	4	>4	0.5
MHB w/ 5 Ca^{2+}/12.5 Mg^{2+} (mg/L)	0.016	0.03	0.016	>4	4	>4	0.5
MHB w/ 50 Ca^{2+}/25 Mg^{2+} (mg/L)	0.03	0.03	0.016	>4	2	>4	0.25
18 hr incubation	0.016 (0.008-0.06)	0.03	0.016	>4	2	>4	0.25 (0.12-1)
24 hr Incubation	0.016	0.03	0.03	>4	2	>4	0.5
48 hr Incubation	0.016	0.03	0.03	>4	4	>4	0.5
no human serum/albumin	0.016 (0.008-0.06)	0.03	0.016	>4	2	>4	0.25 (0.12-1)
10% human serum	0.016	0.03	0.03	NG	>4	>4	0.5
50% human serum	0.016	0.03	0.03	NG	>4	4	0.5
4% human serum albumin	0.03	0.016	0.03	>4	2	>4	0.5
Urine (pH 6.8)	0.016	0.016	0.016	>4	4	>4	0.12
Urine (pH 7.2)	0.008	0.03	0.03	>4	4	>4	0.12
CAMHB (pH 7.2)	0.016 (0.008-0.06)	0.016	0.016	>4	>4	>4	0.25 (0.12-1)
CAMHB (pH 6.8)	0.016	0.016	0.016	>4	4	>4	0.12
3% lysed horse blood	0.016	0.016	0.016	>4	>4	>4	0.25
0.002% P-80	0.016	0.03	0.016	>4	4	>4	0.5
no supplementation	0.016 (0.008-0.06)	0.03	0.016	>4	2	>4	0.25 (0.12-1)

Supplemental Table 1 continued…

Conditions	MIC (µg/mL) Aztreonam						
**Isolate**	***E. coli*** **ATCC 25922**	***E. coli*** **NCTC 13353**	***K. pneumoniae*** **ATCC 700603**	***K. pneumoniae*** **ATCC BAA-1705**	***K. pneumoniae*** **CDC 0135**	***K. pneumoniae*** **ATCC BAA-2146**	***P. aeruginosa*** **ATCC 27853**
**pH 5.0**	0.25	64	32	>64	>64	>64	4
**pH 6.0**	0.12	64	64	>64	>64	>64	2
**pH 7.4**	≤0.06 (0.06-0.25)	64	64 (8-64)	>64	>64	>64	2 (2-8)
**pH 8.0**	≤0.06	64	16	>64	>64	64	2
**Inoculum – 1.1 x 10^4 CFU/mL**	0.12	64	16	>64	>64	64	4
**Inoculum – 1.1 x 10^5 CFU/mL**	0.12 (0.06-0.25)	64	32 (8-64)	>64	>64	>64	4 (2-8)
**Inoculum – 1.1 x 10^6 CFU/mL**	>64#	>64	>64#	>64	>64	>64	64#
**Inoculum – 1.1 x 10^7 CFU/mL**	>64#	>64	>64#	>64	>64	>64	>64#
**ambient atmosphere**	≤0.06 (0.06-0.25)	64	64 (8-64)	>64	>64	>64	2 (2-8)
**5% CO_2 atmosphere**	≤0.06	>64	64	>64	>64	>64	1
**MHB w/ 25 Ca^{2+}/12.5 Mg^{2+} (mg/L)**	0.12 (0.06-0.25)	64	32 (8-64)	>64	>64	64	8 (2-8)
**MHB w/ 5 Ca^{2+}/5 Mg^{2+} (mg/L)**	0.12	64	32	>64	>64	64	4
**MHB w/ 25 Ca^{2+}/5 Mg^{2+} (mg/L)**	0.12	64	32	>64	>64	64	4
**MHB w/ 5 Ca^{2+}/12.5 Mg^{2+} (mg/L)**	0.12	64	32	>64	>64	64	4
**MHB w/ 50 Ca^{2+}/25 Mg^{2+} (mg/L)**	0.12	64	32	>64	>64	>64	4
**18 hr incubation**	≤0.06 (0.06-0.25)	64	64 (8-64)	>64	>64	>64	2 (2-8)
**24 hr Incubation**	≤0.06	64	64	>64	>64	>64	2
**48 hr Incubation**	≤0.06	64	64	>64	>64	>64	2
**no human serum/albumin**	0.12 (0.06-0.25)	64	32 (8-64)	>64	>64	>64	2 (2-8)
**10% human serum**	0.12	64	32	NG	>64	64	2
**50% human serum**	≤0.06	16	16	NG	>64	64	1#
**4% human serum albumin**	0.25	64	64	>64	>64	>64	4
**Urine (pH 6.8)**	≤0.06	64	32	>64	>64	64	0.25#
**Urine (pH 7.2)**	≤0.06	8	16	>64	64	64	0.12#
**CAMHB (pH 7.2)**	≤0.06 (0.06-0.25)	64	64 (8-64)	>64	>64	>64	2 (2-8)
**CAMHB (pH 6.8)**	≤0.06	64	64	>64	>64	>64	2
**3% lysed horse blood**	≤0.06	64	64	>64	>64	64	1#
**0.002% P-80**	≤0.06	>64	32	>64	>64	>64	1#
**no supplementation**	≤0.06 (0.06-0.25)	64	64 (8-64)	>64	>64	>64	2 (2-8)

Supplemental Table 1 continued…

Conditions	MIC (μg/mL)						
	Avibactam						
Isolate	*E. coli* ATCC 25922	*E. coli* NCTC 13353	*K. pneumoniae* ATCC 700603	*K. pneumoniae* ATCC BAA-1705	*K. pneumoniae* CDC 0135	*K. pneumoniae* ATCC BAA-2146	*P. aeruginosa* ATCC 27853
pH 5.0	>128	32	128	64	128	>128	>128
pH 6.0	32	16	32	32	16	32	>128
pH 7.4	4	8	64	16	8	16	>128
pH 8.0	8	16	16	8	16	16	>128
Inoculum – 1.1 x 10^4 CFU/mL	8	8	8	8	8	8	>128
Inoculum – 1.1 x 10^5 CFU/mL	8	8	32	8	128	16	>128
Inoculum – 1.1 x 10^6 CFU/mL	>128	16	128	>128	128	>128	>128
Inoculum – 1.1 x 10^7 CFU/mL	>128	>128	>128	>128	>128	>128	>128
ambient atmosphere	4	8	64	16	8	16	>128
5% CO_2 atmosphere	8	8	32	32	8	8	>128
MHB w/ 25 Ca^{2+}/12.5 Mg^{2+} (mg/L)	16	8	64	8	32	8	>128
MHB w/ 5 Ca^{2+}/5 Mg^{2+} (mg/L)	16	8	16	8	32	8	>128
MHB w/ 25 Ca^{2+}/5 Mg^{2+} (mg/L)	8	32	16	16	64	16	>128
MHB w/ 5 Ca^{2+}/12.5 Mg^{2+} (mg/L)	16	8	32	16	32	8	>128
MHB w/ 50 Ca^{2+}/25 Mg^{2+} (mg/L)	16	16	64	16	128	16	>128
18 hr incubation	4	8	64	16	8	16	>128
24 hr Incubation	8	16	64	64	64	16	>128
48 hr Incubation	128	128	>128	64	64	>128	>128
no human serum/albumin	16	16	32	16	16	16	>128
10% human serum	16	16	32	NG	8	8	>128
50% human serum	32	16	32	NG	128	1	>128
4% human serum albumin	16	8	32	8	16	8	>128
Urine (pH 6.8)	32	8	8	16	16	8	128
Urine (pH 7.2)	16	16	8	16	16	8	64
CAMHB (pH 7.2)	16	8	16	8	32	8	>128
CAMHB (pH 6.8)	16	16	32	16	16	8	>128
3% lysed horse blood	16	8	64	32	8	8	>128
0.002% P-80	16	16	64	16	16	32	>128
no supplementation	4	8	64	16	8	16	>128

Cells shaded grey represent MIC values as observed under standard conditions. CLSI QC ranges shown in parentheses where applicable.
#, denotes where an MIC value fell outside of the CLSI acceptable QC range in a condition for that particular isolate

Supplemental Table 2. Line listing of broth and agar MIC values (µg/mL) and broth:agar MIC ratios for aztreonam/avibactam and aztreonam alone

Organism	Isolate No.	BL Content	Aztreonam (Broth MIC)	Aztreonam (Agar MIC)	Aztreonam Broth: Agar MIC ratio	Aztreonam with Avibactam[1] (Broth MIC)	Aztreonam with Avibactam[1] (Agar MIC)	Aztreonam with Avibactam[1] Broth: Agar MIC ratio
E. coli	ATCC 25922	QC; non-ESBL	0.25	0.12	2	0.12	0.03	4
E. coli	ATCC 35218	TEM-1	0.06	0.12	0.5	0.015	0.03	0.5
E. coli	ATCC BAA-2523	OXA-48	16	8	2	0.12	0.06	2
E. coli	CDC 0048	NDM-1,CMY-6,CTX-M-15,OXA-2,TEM-1B	>256	>256	ID	2	1	2
E. coli	CDC 0055	NDM-1,CMY-6,OXA-1	16	16	1	0.12	0.06	2
E. coli	CDC 0061	KPC-3,OXA-9,TEM-1A	>256	256	>1	0.06	0.06	1
E. coli	CDC 0069	NDM-1,CMY-6,TEM-1B	4	8	0.5	0.06	0.03	2
E. coli	CDC 0114	KPC-3, TEM-1B	>256	128	>2	0.12	0.25	0.5
E. coli	CDC 0118	CMY-6, NDM-1, OXA-2, TEM-1A	32	32	1	4	4	1
E. coli	CDC 0149	NDM-7,CMY-42	1	0.5	2	0.25	0.12	2

Organism	Isolate No.	BL Content	Aztreonam (Broth MIC)	Aztreonam (Agar MIC)	Aztreonam Broth: Agar MIC ratio	Aztreonam with Avibactam[1] (Broth MIC)	Aztreonam with Avibactam[1] (Agar MIC)	Aztreonam with Avibactam[1] Broth: Agar MIC ratio
E. coli	CDC 0150	CMY-42, NDM-5, TEM-1B	32	32	1	4	2	2
E. coli	CDC 0151	NDM-5,CMY-42,CTX-M-15,OXA-1,SHV-12,TEM-1B	>256	>256	ID	2	1	2
E. coli	CDC 0162	CTX-M-15, NDM-7, TEM-1B	>256	128	>2	2	1	2
E. coli	CDC 0369	CTX-M-15	64	32	2	0.03	0.03	1
E. coli	CDC 0435	NDM-1	>256	256	>1	8	4	2
E. coli	CDC 0451	KPC	>256	64	>4	0.12	0.12	1
E. coli	CDC 0452	NDM	0.25	0.12	2	0.06	0.03	2
E. coli	CDC 0503	CTX-M-15; NDM-1; OXA-181	64	64	1	0.06	0.06	1
E. coli	IHMA 1088419	Uncharacterized Clinical Isolate	64	16	4	4	8	0.5
E. coli	IHMA 1123599	Uncharacterized Clinical Isolate	>256	>256	ID	4	8	0.5
E. coli	IHMA 1152602	Uncharacterized Clinical Isolate	64	256	0.25	8	16	0.5

Organism	Isolate No.	BL Content	Aztreonam (Broth MIC)	Aztreonam (Agar MIC)	Aztreonam Broth: Agar MIC ratio	Aztreonam with Avibactam[1] (Broth MIC)	Aztreonam with Avibactam[1] (Agar MIC)	Aztreonam with Avibactam[1] Broth: Agar MIC ratio
E. coli	IHMA 1254068	Uncharacterized Clinical Isolate	>256	>256	ID	4	4	1
E. coli	IHMA 1286157	Uncharacterized Clinical Isolate	32	16	2	2	2	1
E. coli	IHMA 1286214	Uncharacterized Clinical Isolate	>256	256	>1	8	16	0.5
E. coli	IHMA 1413828	Uncharacterized Clinical Isolate	>256	>256	ID	16	16	1
E. coli	IHMA 1491303	Uncharacterized Clinical Isolate	>256	>256	ID	4	8	0.5
E. coli	IHMA 1921199	Uncharacterized Clinical Isolate	0.06	0.06	1	0.06	0.03	2
E. coli	IHMA 1927876	Uncharacterized Clinical Isolate	>256	128	>2	16	16	1
E. coli	IHMA 1932443	Uncharacterized Clinical Isolate	16	32	0.5	8	16	0.5
E. coli	IHMA 1935115	Uncharacterized Clinical Isolate	>256	>256	ID	128	64	2
E. coli	IHMA 1976374	Uncharacterized Clinical Isolate	0.06	0.06	1	0.03	0.004	8
E. coli	IHMA 1999799	Uncharacterized Clinical Isolate	32	32	1	8	8	1

Organism	Isolate No.	BL Content	Aztreonam (Broth MIC)	Aztreonam (Agar MIC)	Aztreonam Broth: Agar MIC ratio	Aztreonam with Avibactam[1] (Broth MIC)	Aztreonam with Avibactam[1] (Agar MIC)	Aztreonam with Avibactam[1] Broth: Agar MIC ratio
E. coli	IHMA 2059576	Uncharacterized Clinical Isolate	>256	>256	ID	8	16	0.5
E. coli	IHMA 2060289	Uncharacterized Clinical Isolate	>256	>256	ID	8	8	1
E. coli	MMX 1391	Non-ESBL	0.06	0.06	1	0.015	0.03	0.5
E. coli	MMX 2214	Non-ESBL	0.12	0.12	1	0.06	0.06	1
E. coli	MMX 2234	DHA; SHV; TEM	256	128	2	0.5	0.25	2
E. coli	MMX 2246	Non-ESBL	0.12	0.12	1	0.06	0.06	1
E. coli	MMX 2247	Non-ESBL	0.03	0.06	0.5	0.03	0.03	1
E. coli	MMX 2269	CTX-M-1; TEM	32	8	4	0.06	0.06	1
E. coli	MMX 2504	CTX-M-1; OXA-1; SHV	256	128	2	1	0.5	2
E. coli	MMX 2506	CTX-M-1; OXA-1; TEM	32	32	1	0.06	0.06	1
E. coli	MMX 2513	SVH; TEM	256	128	2	0.12	0.25	0.5

Organism	Isolate No.	BL Content	Aztreonam (Broth MIC)	Aztreonam (Agar MIC)	Aztreonam Broth: Agar MIC ratio	Aztreonam with Avibactam[1] (Broth MIC)	Aztreonam with Avibactam[1] (Agar MIC)	Aztreonam with Avibactam[1] Broth: Agar MIC ratio
E. coli	MMX 5300	Non-ESBL	0.008	0.015	0.5	0.015	0.015	1
E. coli	MMX 5391	CTX-M-1	64	32	2	0.06	0.06	1
E. coli	MMX 5741	CTX-M-1; OXA-1; OXA-9; TEM	32	16	2	0.06	0.06	1
E. coli	MMX 5743	CTX-M-1; OXA-1; TEM	128	64	2	0.25	0.12	2
E. coli	MMX 5744	CTX-M-1; OXA-1	128	64	2	0.12	0.12	1
E. coli	MMX 5745	KPC; TEM	256	64	4	0.12	0.06	2
E. coli	MMX 5746	CTX-M-1; OXA-1; SHV	64	32	2	0.06	0.06	1
E. coli	MMX 5751	SHV; TEM	>256	256	>1	1	0.5	2
E. coli	MMX 5756	OXA-9; SHV; TEM	256	64	4	0.12	0.12	1
E. coli	MMX 5758	CTX-M-1; SHV; TEM	32	32	1	0.06	0.03	2
E. coli	MMX 5759	CMY-2; CMY-41; OXA-1; OXA-9; SHV; TEM	32	32	1	0.5	0.25	2

Organism	Isolate No.	BL Content	Aztreonam (Broth MIC)	Aztreonam (Agar MIC)	Aztreonam Broth: Agar MIC ratio	Aztreonam with Avibactam[1] (Broth MIC)	Aztreonam with Avibactam[1] (Agar MIC)	Aztreonam with Avibactam[1] Broth: Agar MIC ratio
E. coli	MMX 5762	OXA-9; SHV; TEM	64	32	2	0.06	0.12	0.5
E. coli	MMX 5768	CTX-M-1; OXA-1; SHV; TEM	64	32	2	0.06	0.06	1
E. coli	NCTC 13353	CTX-M-15	>256	64	>4	0.06	0.03	2
K. pneumoniae	ATCC 700603	SHV-18; OXA-2	64	32	2	0.12	0.25	0.5
K. pneumoniae	ATCC BAA-1705	KPC-2; SHV; TEM	>256	>256	ID	0.25	0.12	2
K. pneumoniae	CDC 0034	IMP-4, SHV-11, TEM-1B	0.06	0.12	0.5	0.06	0.06	1
K. pneumoniae	CDC 0040	VIM-27,CTX-M-15,OXA-1,SHV-11	256	64	4	0.12	0.12	1
K. pneumoniae	CDC 0042	CTX-M-15,OXA-1,OXA-10,SHV-1,TEM-1B	256	64	4	0.25	0.12	2
K. pneumoniae	CDC 0043	SHV-12	>256	256	>1	0.25	0.25	1
K. pneumoniae	CDC 0044	CTX-M-15,OXA-1,OXA-9,SHV-12,TEM-1A	256	256	1	0.25	0.12	2
K. pneumoniae	CDC 0046	VIM-27,CTX-M-15,OXA-1,SHV-11	128	128	1	0.12	0.12	1

Organism	Isolate No.	BL Content	Aztreonam (Broth MIC)	Aztreonam (Agar MIC)	Aztreonam Broth: Agar MIC ratio	Aztreonam with Avibactam[1] (Broth MIC)	Aztreonam with Avibactam[1] (Agar MIC)	Aztreonam with Avibactam[1] Broth: Agar MIC ratio
K. pneumoniae	CDC 0049	NDM-1,CMY-6,CTX-M-15,OXA-1,TEM-1B	128	128	1	0.25	0.12	2
K. pneumoniae	CDC 0066	OXA-232,CTX-M-15,OXA-1,OXA-9,TEM-1A	256	256	1	0.5	0.5	1
K. pneumoniae	CDC 0068	NDM-1,OXA-232,CTX-M-15,OXA-1,OXA-9,SHV-11,TEM-1A	256	256	1	0.5	0.5	1
K. pneumoniae	CDC 0075	OXA-232,CTX-M-15,OXA-1,SHV-1	256	256	1	0.25	0.12	2
K. pneumoniae	CDC 0076	VIM-1,SHV-30	0.5	0.5	1	0.06	0.06	1
K. pneumoniae	CDC 0080	IMP-4, IKP-B-2, OXA-1, SFO-1, TEM-1B	64	64	1	0.25	0.25	1
K. pneumoniae	CDC 0438	KPC	>256	>256	ID	1	0.5	2
K. pneumoniae	CDC 0453	KPC	>256	>256	ID	2	2	1
K. pneumoniae	CDC 0504	SHV-OSBL(b); CTX-M-15; OXA-48	32	64	0.5	0.06	0.12	0.5
K. pneumoniae	CDC 0505	SHV-12(e); TEM-OSBL(b); CTX-M-15; NDM-1	32	16	2	0.03	0.03	1
K. pneumoniae	CDC 0507	SHV-OSBL(b); TEM-OSBL(b); CTX-M-15; NDM-1; OXA-232	256	128	2	1	0.5	2

Organism	Isolate No.	BL Content	Aztreonam (Broth MIC)	Aztreonam (Agar MIC)	Aztreonam Broth: Agar MIC ratio	Aztreonam with Avibactam[1] (Broth MIC)	Aztreonam with Avibactam[1] (Agar MIC)	Aztreonam with Avibactam[1] Broth: Agar MIC ratio
K. pneumoniae	CDC 0522	KPC-2	>256	>256	ID	0.12	0.12	1
K. pneumoniae	CDC 0523	KPC-2	>256	256	>1	0.12	0.12	1
K. pneumoniae	CDC 0524	SHV-12; KPC-3	>256	>256	ID	0.5	0.5	1
K. pneumoniae	IHMA 1319427	Uncharacterized Clinical Isolate	>256	>256	ID	8	8	1
K. pneumoniae	IHMA 2009789	Uncharacterized Clinical Isolate	>256	>256	ID	64	8	8
K. pneumoniae	IHMA 2111864	Uncharacterized Clinical Isolate	256	64	4	0.06	0.03	2
K. pneumoniae	MMX 2238	Non-ESBL	2	1	2	0.03	0.06	0.5
K. pneumoniae	MMX 4382	Non-ESBL	1	0.5	2	0.5	0.25	2
K. pneumoniae	MMX 6855	CMY-2	128	64	2	2	1	2
K. pneumoniae	MMX 6856	MIR-1	64	64	1	1	1	1
K. pneumoniae	MMX 6858	TEM-4	32	32	1	0.25	0.25	1

Organism	Isolate No.	BL Content	Aztreonam (Broth MIC)	Aztreonam (Agar MIC)	Aztreonam Broth: Agar MIC ratio	Aztreonam with Avibactam[1] (Broth MIC)	Aztreonam with Avibactam[1] (Agar MIC)	Aztreonam with Avibactam[1] Broth: Agar MIC ratio
K. pneumoniae	MMX 6859	TEM-12	8	4	2	0.25	0.25	1
K. pneumoniae	MMX 6862	SHV-5	>256	256	>1	1	0.5	2
K. pneumoniae	MMX 6864	CTX-M-15	32	32	1	0.5	0.25	2
K. pneumoniae	MMX 6866	OXA-10	16	8	2	1	0.5	2
K. pneumoniae	MMX 8706	KPC, SHV, TEM	>256	256	>1	0.25	0.12	2
K. pneumoniae	MMX 9033	Non-ESBL	64	16	4	0.12	0.12	1
K. pneumoniae	MMX 9059	CTX-M-1, SHV	>256	64	>4	0.25	0.25	1
K. pneumoniae	MMX 9060	CTX-M-1, KPC, SHV	128	64	2	0.12	0.12	1
K. pneumoniae	MMX 9061	CTX-M-1, KPC, SHV, TEM	>256	256	>1	0.25	0.25	1
K. pneumoniae	MMX 9062	CTX-M-1, OXA-1, SHV, TEM	128	128	1	0.12	0.12	1
K. pneumoniae	MMX 9255	CTX-M-1, OXA-1, OXA-9, SHV, TEM	>256	128	>2	0.12	0.12	1

Organism	Isolate No.	BL Content	Aztreonam (Broth MIC)	Aztreonam (Agar MIC)	Aztreonam Broth: Agar MIC ratio	Aztreonam with Avibactam[1] (Broth MIC)	Aztreonam with Avibactam[1] (Agar MIC)	Aztreonam with Avibactam[1] Broth: Agar MIC ratio
K. pneumoniae	MMX 9263	KPC, OXA-1, OXA-9, SHV, TEM	>256	>256	ID	0.25	0.12	2
K. pneumoniae	MMX 9302	CTX-M-15, OXA-1, SHV, TEM	32	32	1	0.06	0.06	1
K. pneumoniae	MMX 9303	CTX-M-15, OXA-1,OXA-9, SHV, TEM	16	16	1	0.03	0.03	1
K. pneumoniae	MMX 9304	CTX-M-15, OXA-1, SHV	32	64	0.5	0.03	0.06	0.5
K. pneumoniae	MMX 9305	OXA-1, SHV	16	16	1	0.12	0.12	1
K. pneumoniae	MMX 9306	CTX-M-15, SHV	128	128	1	0.25	0.12	2
K. pneumoniae	NCTC 13440	VIM-1	256	128	2	0.12	0.06	2
K. aerogenes	IHMA 2024689	Uncharacterized Clinical Isolate	>256	256	>1	8	4	2
K. aerogenes	MMX 6080	Uncharacterized Clinical Isolate	0.25	0.03	8	0.12	0.06	2
K. aerogenes	MMX 6081	Uncharacterized Clinical Isolate	0.25	0.06	4	0.12	0.06	2
K. aerogenes	MMX 6082	Uncharacterized Clinical Isolate	128	16	8	0.5	0.12	4

Organism	Isolate No.	BL Content	Aztreonam (Broth MIC)	Aztreonam (Agar MIC)	Aztreonam Broth: Agar MIC ratio	Aztreonam with Avibactam[1] (Broth MIC)	Aztreonam with Avibactam[1] (Agar MIC)	Aztreonam with Avibactam[1] Broth: Agar MIC ratio
E. cloacae	ATCC BAA-1143	ampC	128	128	1	2	1	2
E. cloacae	CDC 0038	NDM-1,ACT-7,CTX-M-15,OXA-1,OXA-9,TEM-1B	>256	128	>2	1	0.5	2
E. cloacae	CDC 0050	KPC-4,ACT-5,TEM-1A	256	256	1	0.5	0.25	2
E. cloacae	CDC 0053	KPC-3,OXA-9,TEM-1A	>256	256	>1	0.25	0.12	2
E. cloacae	CDC 0132	NMC-A	16	2	8	0.12	0.06	2
E. cloacae	CDC 0136	KPC-3, OXA-9, SVH-12, TEM-1A	>256	256	>1	0.25	0.25	1
E. cloacae	CDC 0154	TEM-1B, VIM-1	64	64	1	0.12	0.06	2
E. cloacae	CDC 0163	CTX-M-15, KPC-2, OXA-1, TEM-1B	>256	128	>2	0.12	0.12	1
E. cloacae	CDC 0164	NMC-A	>256	128	>2	1	1	1
E. cloacae	CDC 0366	KPC	>256	128	>2	0.25	0.25	1
E. cloacae	CDC 0448	NDM	128	128	1	16	8	2

Organism	Isolate No.	BL Content	Aztreonam (Broth MIC)	Aztreonam (Agar MIC)	Aztreonam Broth: Agar MIC ratio	Aztreonam with Avibactam[1] (Broth MIC)	Aztreonam with Avibactam[1] (Agar MIC)	Aztreonam with Avibactam[1] Broth: Agar MIC ratio
E. cloacae	CDC 0501	TEM-OSBL, CTX-M-9, VIM-1	2	2	1	0.25	0.06	4
E. cloacae	CDC 0502	IMP-8, SHV-12, TEM-1B	256	128	2	0.5	0.5	1
E. cloacae	IHMA 1082734	Uncharacterized Clinical Isolate	>256	>256	ID	8	8	1
E. cloacae	IHMA 1100876	Uncharacterized Clinical Isolate	>256	128	>2	4	8	0.5
E. cloacae	IHMA 1261347	Uncharacterized Clinical Isolate	>256	>256	ID	32	16	2
E. cloacae	IHMA 1832049	Uncharacterized Clinical Isolate	256	256	1	16	8	2
E. cloacae	IHMA 2026111	Uncharacterized Clinical Isolate	32	32	1	4	4	1
E. cloacae	IHMA 2111866	Uncharacterized Clinical Isolate	16	0.5	32	0.25	0.06	4
E. cloacae	MMC 10832	KPC	>256	64	>4	0.06	0.06	1
E. cloacae	MMC 10835	KPC	>256	256	>1	4	2	2
E. cloacae	MMC 10839	VIM	32	32	1	0.5	0.5	1

Organism	Isolate No.	BL Content	Aztreonam (Broth MIC)	Aztreonam (Agar MIC)	Aztreonam Broth: Agar MIC ratio	Aztreonam with Avibactam[1] (Broth MIC)	Aztreonam with Avibactam[1] (Agar MIC)	Aztreonam with Avibactam[1] Broth: Agar MIC ratio
E. cloacae	MMX 10830	IMP	2	1	2	0.5	0.25	2
E. cloacae	MMX 10831	KPC	32	64	0.5	1	0.5	2
E. cloacae	MMX 10833	KPC	>256	64	>4	0.06	0.06	1
E. cloacae	MMX 10834	KPC	>256	256	>1	0.25	0.12	2
E. cloacae	MMX 10838	VIM	0.12	0.06	2	0.03	0.06	0.5
E. cloacae	MMX 10840	VIM	32	32	1	0.5	0.5	1
E. cloacae	MMX 10841	VIM	32	32	1	0.5	0.5	1
E. asburiae	IHMA 2111873	Uncharacterized Clinical Isolate	0.12	0.12	1	0.06	0.06	1
E. bugandensis	IHMA 2111867	Uncharacterized Clinical Isolate	0.06	0.06	1	0.06	0.03	2
C. freundii	CDC 0116	CMY-76, CMY-79, KPC-2	>256	256	>1	0.5	0.25	2
C. freundii	CDC 0157	CTX-M-15, NDM-1, OXA-9, TEM-1B	128	128	1	0.5	0.25	2

Organism	Isolate No.	BL Content	Aztreonam (Broth MIC)	Aztreonam (Agar MIC)	Aztreonam Broth: Agar MIC ratio	Aztreonam with Avibactam[1] (Broth MIC)	Aztreonam with Avibactam[1] (Agar MIC)	Aztreonam with Avibactam[1] Broth: Agar MIC ratio
C. freundii	MMX 10856	KPC	256	64	4	0.25	0.25	1
C. freundii	MMX 10857	NDM	>256	32	>8	0.06	0.06	1
C. freundii	MMX 10858	KPC	64	8	8	0.06	0.03	2
C. freundii	MMX 10859	KPC	>256	32	>8	0.03	0.03	1
C. freundii	MMX 10860	KPC	>256	128	>2	0.12	0.12	1
C. freundii	MMX 10861	KPC	>256	8	>32	0.06	0.06	1
C. freundii	MMX 10862	KPC	128	16	8	0.03	0.06	0.5
C. freundii	MMX 8414	Uncharacterized Clinical Isolate	0.12	0.06	2	0.06	0.06	1
C. koseri	MMX 10879	Uncharacterized Clinical Isolate	128	64	2	0.12	0.12	1
C. koseri	MMX 6068	Uncharacterized Clinical Isolate	0.03	0.03	1	0.03	0.03	1
C. koseri	MMX 6069	Uncharacterized Clinical Isolate	0.25	0.25	1	0.12	0.12	1

Organism	Isolate No.	BL Content	Aztreonam (Broth MIC)	Aztreonam (Agar MIC)	Aztreonam Broth: Agar MIC ratio	Aztreonam with Avibactam[1] (Broth MIC)	Aztreonam with Avibactam[1] (Agar MIC)	Aztreonam with Avibactam[1] Broth: Agar MIC ratio
C. koseri	MMX 6070	Uncharacterized Clinical Isolate	0.25	0.25	1	0.12	0.12	1
C. koseri	MMX 6282	Uncharacterized Clinical Isolate	0.12	0.06	2	0.06	0.03	2
C. koseri	MMX 6360	Uncharacterized Clinical Isolate	0.06	0.06	1	0.03	0.06	0.5
C. koseri	MMX 6362	Uncharacterized Clinical Isolate	0.06	0.06	1	0.03	0.03	1
C. koseri	MMX 6367	Uncharacterized Clinical Isolate	0.06	0.06	1	0.06	0.06	1
P. mirabilis	CDC 0155	KPC-6	>256	16	>16	0.015	0.008	2
P. mirabilis	CDC 0156	KPC-2, OXA-10	0.25	0.015	16	0.008	0.008	1
P. mirabilis	CDC 0159	NDM-1	0.008	0.008	1	0.008	0.008	1
P. mirabilis	CDC 0377	KPC	>256	1	>256	0.008	0.008	1
P. mirabilis	MMX 10559	Uncharacterized Clinical Isolate	0.015	0.008	2	0.008	0.008	1
P. mirabilis	MMX 10561	Uncharacterized Clinical Isolate	0.008	0.008	1	0.008	0.008	1

Organism	Isolate No.	BL Content	Aztreonam (Broth MIC)	Aztreonam (Agar MIC)	Aztreonam Broth: Agar MIC ratio	Aztreonam with Avibactam[1] (Broth MIC)	Aztreonam with Avibactam[1] (Agar MIC)	Aztreonam with Avibactam[1] Broth: Agar MIC ratio
P. mirabilis	MMX 10565	Uncharacterized Clinical Isolate	0.06	0.015	4	0.008	0.008	1
P. mirabilis	MMX 10699	Uncharacterized Clinical Isolate	0.008	0.008	1	0.008	0.015	0.5
P. mirabilis	MMX 10703	Uncharacterized Clinical Isolate	0.008	0.008	1	0.008	0.008	1
P. mirabilis	MMX 11058	Uncharacterized Clinical Isolate	0.12	0.25	0.5	0.008	0.008	1
P. vulgaris	MMC 8479	Uncharacterized Clinical Isolate	0.03	0.015	2	0.015	0.015	1
P. vulgaris	MMX 8476	Uncharacterized Clinical Isolate	0.12	0.008	16	0.008	0.008	1
P. vulgaris	MMX 8485	Uncharacterized Clinical Isolate	0.015	0.008	2	0.008	0.004	2
P. vulgaris	MMX 8576	Uncharacterized Clinical Isolate	0.06	0.015	4	0.015	0.03	0.5
P. vulgaris	MMX 8579	Uncharacterized Clinical Isolate	2	0.008	256	0.015	0.008	2
P. vulgaris	MMX 8585	Uncharacterized Clinical Isolate	0.015	0.015	1	0.015	0.008	2
P. vulgaris	MMX 9163	Uncharacterized Clinical Isolate	0.25	0.015	16	0.015	0.008	2

Organism	Isolate No.	BL Content	Aztreonam (Broth MIC)	Aztreonam (Agar MIC)	Aztreonam Broth: Agar MIC ratio	Aztreonam with Avibactam[1] (Broth MIC)	Aztreonam with Avibactam[1] (Agar MIC)	Aztreonam with Avibactam[1] Broth: Agar MIC ratio
P. vulgaris	MMX 9261	Uncharacterized Clinical Isolate	256	0.015	16384	0.008	0.004	2
P. vulgaris	MMX 9276	Uncharacterized Clinical Isolate	0.015	0.015	1	0.015	0.008	2
P. vulgaris	MMX 9373	Uncharacterized Clinical Isolate	0.25	0.12	2	0.25	0.12	2
S. marcescens	CDC 0520	FOX-5	2	2	1	0.12	0.12	1
S. marcescens	CDC 0521	CMY-16	256	128	2	1	0.5	2
S. marcescens	MMX 8562	Uncharacterized Clinical Isolate	0.12	0.12	1	0.25	0.25	1
S. marcescens	MMX 8565	Uncharacterized Clinical Isolate	2	0.25	8	0.25	0.25	1
S. marcescens	MMX 9152	Uncharacterized Clinical Isolate	0.12	0.25	0.5	0.12	0.25	0.5
S. marcescens	MMX 9155	Uncharacterized Clinical Isolate	0.25	0.12	2	0.25	0.12	2
S. marcescens	MMX 9271	Uncharacterized Clinical Isolate	0.12	0.5	0.25	0.12	0.12	1
M. morganii	CDC 0519	DHA-TYPE	32	32	1	0.5	0.25	2

Organism	Isolate No.	BL Content	Aztreonam (Broth MIC)	Aztreonam (Agar MIC)	Aztreonam Broth: Agar MIC ratio	Aztreonam with Avibactam[1] (Broth MIC)	Aztreonam with Avibactam[1] (Agar MIC)	Aztreonam with Avibactam[1] Broth: Agar MIC ratio
M. morganii	IHMA 1072800	Uncharacterized Clinical Isolate	0.015	0.015	1	0.015	0.008	2
M. morganii	MMX 6429	Uncharacterized Clinical Isolate	0.06	0.03	2	0.015	0.015	1
M. morganii	MMX 6431	Uncharacterized Clinical Isolate	0.06	0.12	0.5	0.015	0.015	1
M. morganii	MMX 6572	Uncharacterized Clinical Isolate	2	4	0.5	0.06	0.12	0.5
M. morganii	MMX 6609	Uncharacterized Clinical Isolate	0.015	0.12	0.12	0.015	0.008	2
M. morganii	MMX 6611	Uncharacterized Clinical Isolate	0.03	0.008	4	0.015	0.015	1
M. morganii	MMX 6613	Uncharacterized Clinical Isolate	0.25	0.12	2	0.015	0.015	1
P. rettgeri	IHMA 1270260	Uncharacterized Clinical Isolate	16	32	0.5	16	32	0.5
P. rettgeri	MMX 8504	Uncharacterized Clinical Isolate	0.03	0.03	1	0.03	0.03	1
P. rettgeri	MMX 8596	Uncharacterized Clinical Isolate	0.004	0.004	1	0.004	0.004	1
P. stuartii	MMX 8511	Uncharacterized Clinical Isolate	0.5	0.008	64	0.008	0.008	1

Organism	Isolate No.	BL Content	Aztreonam (Broth MIC)	Aztreonam (Agar MIC)	Aztreonam Broth: Agar MIC ratio	Aztreonam with Avibactam[1] (Broth MIC)	Aztreonam with Avibactam[1] (Agar MIC)	Aztreonam with Avibactam[1] Broth: Agar MIC ratio
P. stuartii	MMX 8611	Uncharacterized Clinical Isolate	0.12	0.015	8	0.03	0.015	2
P. stuartii	MMX 8614	Uncharacterized Clinical Isolate	0.03	0.008	4	0.015	0.03	0.5
P. stuartii	MMX 9376	Uncharacterized Clinical Isolate	0.03	0.06	0.5	0.015	0.015	1
R. ornithinolytica	IHMA 1256861	Uncharacterized Clinical Isolate	>256	256	>1	32	32	1

BL: beta-lactamase content, ID: indeterminate

[1] Reported MIC represents the aztreonam concentration of the combination (avibactam tested at constant concentration of 4 µg/mL by both methods)

cells shaded grey indicate discordance (MIC value variation of >2-fold) between methods

Supplemental Table 3. Broth and agar MIC values for aztreonam and aztreonam/avibactam against QC isolates

Isolate	Run No.	MIC (µg/mL)			
		**Aztreonam (Broth MIC)**	**Aztreonam (Agar MIC)**	**Aztreonam with Avibactam[1] (Broth MIC)**	**Aztreonam with Avibactam[1] (Agar MIC)**
E. coli ATCC 25922	1	0.25 (0.06-0.25)[2]	0.12 (0.06-0.25)	0.12 (0.03-0.12)	0.03 (0.03-0.12)
	2	0.25	0.12	0.12	0.06
	3	0.12	0.12	0.25	0.03
	4	0.12	0.12	0.12	0.06
	5	0.25	0.25	0.12	0.06
	6	0.12	0.12	0.12	0.03
	7	0.12	0.06	0.06	0.06
	8	0.25	0.12	0.12	0.06
K. pneumoniae ATCC 700603	1	64 (8-64)	32 (8-64)	0.12 (0.06-0.5)	0.25 (0.06-0.5)
	2	32	32	0.12	0.12
	3	32	32	0.12	0.12
	4	64	32	0.25	0.25
	5	53	32	0.25	0.12
	6	32	32	0.12	0.12
	7	32	16	0.12	0.12
	8	64	32	0.25	0.12

[1] Reported MIC represents the aztreonam concentration of the combination (avibactam tested at constant concentration of 4 µg/mL by both methods)
[2] CLSI QC ranges shown in parentheses where applicable

Supplemental Table 4. Broth and agar MIC values over multiple runs for isolates with initial discordant aztreonam/avibactam MIC values

Organism	Isolate No.	Agar or Broth	RUN 1 (initial test)		RUN 2		RUN 3		RUN 4		RUN 5		Median	
			AZT	AZT/AVI	AZT	AZT/AVI	AZT	AZT/AVI	AZT	AZT/AVI	AZT	AZT/AVI	AZT	AZT/AVI
E. coli	ATCC 25922	Broth	0.25	0.12	0.5	0.12	0.25	0.12	0.12	0.12	0.25	0.12	0.25	0.12
		Agar	0.12	0.03	0.06	0.016	0.12	0.03	0.12	0.06	0.12	0.06	0.12	0.03
E. coli	CDC 0048	Broth	>256	2	>256	1	>256	4	>256	2	>256	2	>256	2
		Agar	>256	0.25	>256	2	>256	1	>256	2	>256	1	>256	1
E. coli	CDC 0055	Broth	16	0.25	16	0.25	16	0.12	16	0.12	8	0.12	16	0.12
		Agar	16	0.06	16	0.016	16	0.06	32	0.12	8	0.06	16	0.06
E. coli	CDC 0069	Broth	4	0.25	4	0.06	4	0.06	8	0.06	4	0.12	4	0.06
		Agar	4	0.03	4	0.03	8	0.03	8	0.06	8	0.03	8	0.03
E. coli	CDC 0150	Broth	64	16	32	4	32	4	32	4	64	4	32	4
		Agar	32	4	32	2	32	1	32	2	32	2	32	2
E. coli	IHMA 1935115	Broth	>256	>256	>256	64	>256	128	>256	64	>256	256	>256	128
		Agar	256	64	>256	64	>256	32	>256	64	>256	64	>256	64
E. coli	IHMA 1976374	Broth	0.06	0.03	0.06	0.03	0.06	0.03	0.06	0.03	0.12	0.06	0.06	0.03
		Agar	0.06	≤0.002	0.06	0.004	0.06	0.008	0.06	0.004	0.12	0.016	0.06	0.004
E. coli	MMX 5743	Broth	128	0.12	128	0.25	128	0.25	128	0.25	128	0.25	128	0.25
		Agar	64	0.5	64	0.12	64	0.12	128	0.12	128	0.12	64	0.12
K. pneumoniae	CDC 0076	Broth	1	0.06	0.5	0.06	0.5	0.06	0.5	0.06	0.5	0.06	0.5	0.06
		Agar	0.25	2	0.5	0.03	0.5	0.06	0.5	0.06	0.5	0.06	0.5	0.06
K. pneumoniae	IHMA 2009789	Broth	>256	64	>256	64	>256	64	>256	64	>256	64	>256	64
		Agar	>256	8	>256	8	>256	8	>256	16	>256	16	>256	8
K. pneumoniae	MMX 6864	Broth	64	2	32	0.25	32	1	64	0.5	32	0.5	32	0.5
		Agar	32	0.25	32	0.25	32	0.25	64	0.25	64	0.25	32	0.25
		Broth	16	0.5	16	0.12	8	0.12	16	0.12	8	0.12	16	0.12

Organism	Isolate No.	Agar or Broth	RUN 1 (initial test)		RUN 2		RUN 3		RUN 4		RUN 5		Median	
			AZT	AZT/AVI	AZT	AZT/AVI	AZT	AZT/AVI	AZT	AZT/AVI	AZT	AZT/AVI	AZT	AZT/AVI
K. pneumoniae	MMX 9305	Agar	16	0.12	16	0.06	16	0.12	16	0.12	16	0.12	16	0.12
K. pneumoniae	NCTC 13440	Broth	128	0.25	256	0.12	256	0.06	128	0.25	256	0.12	256	0.12
		Agar	128	0.06	64	0.06	128	0.06	128	0.06	128	0.06	128	0.06
E. cloacae	CDC 0050	Broth	256	0.5	256	0.5	256	0.25	256	0.25	>256	0.5	256	0.5
		Agar	256	0.12	256	0.25	256	0.12	256	0.25	256	0.25	256	0.25
E. cloacae	CDC 0501	Broth	1	0.25	2	0.12	2	0.25	4	0.25	2	0.25	2	0.25
		Agar	2	0.06	2	0.06	4	0.06	4	0.12	2	0.12	2	0.06
E. cloacae	CDC 0502	Broth	256	2	256	1	256	0.5	256	0.5	256	0.5	256	0.5
		Agar	128	0.5	128	0.5	128	0.5	128	0.5	128	0.5	128	0.5
E. cloacae	IHMA 2111866	Broth	16	0.25	8	0.12	16	0.25	8	0.25	16	0.12	16	0.25
		Agar	0.25	0.06	2	0.06	4	0.06	0.5	0.06	0.5	0.03	0.5	0.06
C. freundi	CDC 0157	Broth	128	0.5	128	0.5	128	0.5	256	0.5	256	0.5	128	0.5
		Agar	64	0.015	64	0.25	128	0.25	128	0.25	128	0.06	128	0.25
P. rettgeri	8504	Broth	128	128	0.03	0.03	0.03	0.03	0.03	0.03	0.03	0.03	0.03	0.03
		Agar	0.015	0.03	0.03	0.03	0.06	0.03	0.03	0.06	0.03	0.06	0.03	0.03
P. rettgeri	8596	Broth	32	32	0.004	0.004	0.004	0.004	0.004	0.004	0.004	0.004	0.004	0.004
		Agar	0.008	0.004	0.004	0.004	0.004	0.004	0.004	0.008	0.008	0.008	0.004	0.004

AZT, aztreonam; AVI, avibactam; No., number

Supplemental Table 5. Analysis of additional *Providencia* spp. by agar and broth

Organism	MMX No.	Aztreonam (Broth MIC)	Aztreonam (Agar MIC)	Aztreonam Broth:Agar MIC ratio	Aztreonam with Avibactam[1] (Broth MIC)	Aztreonam with Avibactam[1] (Agar MIC)	Aztreonam with Avibactam[1] Broth:Agar MIC ratio
P. rettgeri	8496	0.004	0.004	1	0.004	0.004	1
P. rettgeri	8497	0.008	0.008	1	0.008	0.008	1
P. rettgeri	8498	0.004	0.004	1	0.004	0.008	0.5
P. rettgeri	8499	0.004	0.008	0.5	0.004	0.008	0.5
P. rettgeri	8500	0.008	0.008	1	0.008	0.008	1
P. rettgeri	8501	0.004	0.004	1	0.004	0.004	1
P. rettgeri	8502	0.004	0.004	1	0.004	0.004	1
P. rettgeri	8503	0.004	0.004	1	0.004	0.004	1
P. rettgeri	8505	0.004	0.004	1	0.004	0.004	1
P. rettgeri	8597	0.004	0.004	1	0.004	0.004	1
P. rettgeri	8598	0.008	0.016	0.5	0.008	0.016	0.5
P. stuartii	8506	0.03	0.016	2	0.016	0.008	2
P. stuartii	8507	0.03	0.016	2	0.016	0.008	2
P. stuartii	8508	0.016	0.016	1	0.016	0.008	2

Organism	MMX No.	Aztreonam (Broth MIC)	Aztreonam (Agar MIC)	Aztreonam Broth:Agar MIC ratio	Aztreonam with Avibactam[1] (Broth MIC)	Aztreonam with Avibactam[1] (Agar MIC)	Aztreonam with Avibactam[1] Broth:Agar MIC ratio
P. stuartii	8509	0.06	0.06	1	0.016	0.016	1
P. stuartii	8510	0.016	0.03	0.5	0.008	0.008	1
P. stuartii	8513	0.016	0.008	2	0.016	0.008	2
P. stuartii	8514	0.016	0.03	0.5	0.016	0.016	1
P. stuartii	8515	0.03	0.016	2	0.016	0.016	1
P. stuartii	8606	2	4	0.5	0.03	0.03	1
P. stuartii	8607	2	2	1	0.03	0.03	1

[1] Reported MIC represents the aztreonam concentration of the combination (avibactam tested at constant concentration of 4 µg/mL by both methods)

Supplemental Figure 1. ATM/AVI broth microdilution MIC endpoints when testing against *Providencia rettgeri*. ATM/AVI concentration is across double rows of evaluation with the lower row having a lower concentration of drug and the higher concentration of drug on the upper row. ATM/AVI concentration is diluted 1:1 across the row from left to right.

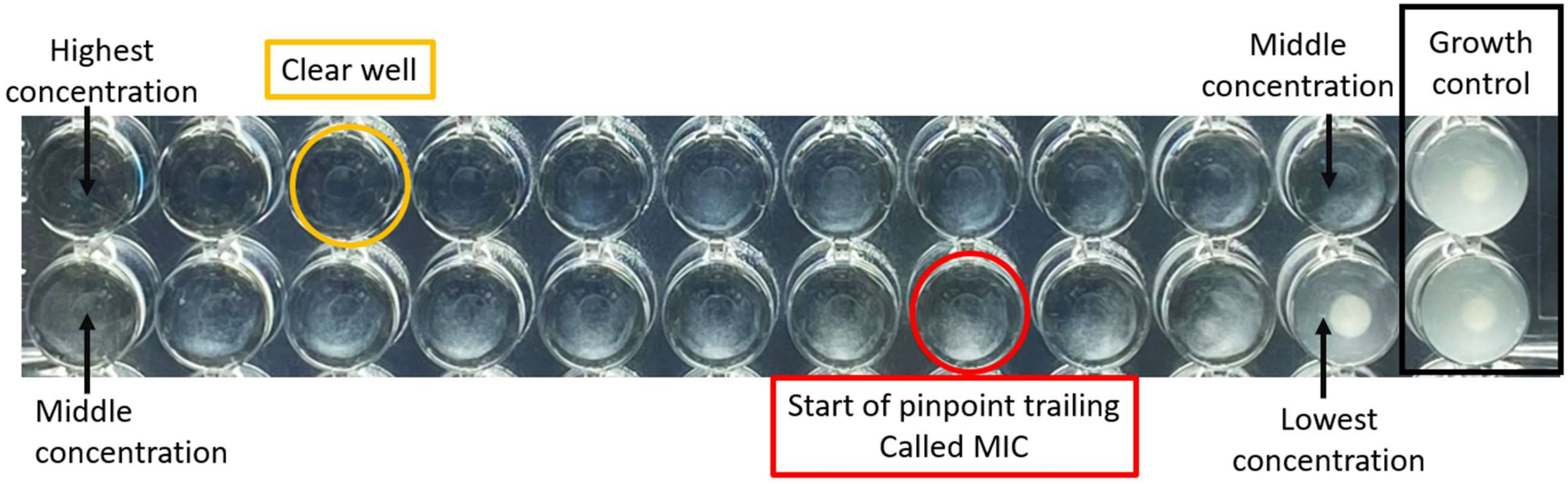

Supplement: Supplemental file 1 — Supplemental material. Download spectrum.03601-22-s0001.pdf, PDF file, 0.8 MB [file spectrum.03601-22-s0001.pdf]
